# The Prevention of Postmenopausal Osteoporotic Fractures: Results of the Health Technology Assessment of a New Antiosteoporotic Drug

**DOI:** 10.1155/2014/975927

**Published:** 2014-02-04

**Authors:** Chiara de Waure, Maria Lucia Specchia, Chiara Cadeddu, Silvio Capizzi, Stefano Capri, Maria Luisa Di Pietro, Maria Assunta Veneziano, Maria Rosaria Gualano, Flavia Kheiraoui, Giuseppe La Torre, Nicola Nicolotti, Antonella Sferrazza, Walter Ricciardi

**Affiliations:** Research Centre for Health Technology Assessment, Department of Public Health, Section of Hygiene, Universitá Cattolica del Sacro Cuore, Largo F. Vito 1, 00168 Rome, Italy

## Abstract

*Objective*. The Health Technology Assessment (HTA) approach was applied to denosumab in the prevention of osteoporotic fractures in postmenopausal women. *Method*. Epidemiological, clinical, technical, economic, organizational, and ethical aspects were considered. Medical electronic databases were accessed to evaluate osteoporosis epidemiology and therapeutical approaches. A budget impact and a cost-effectiveness analyses were performed to assess economic implications. Clinical benefits and patient needs were considered with respect to organizational and ethical evaluation. *Results*. In Italy around four millions women are affected by osteoporosis and have a higher risk for fractures with 70,000 women being hospitalized every year. Bisphosphonates and strontium ranelate are recommended as first line treatment for the prevention of osteoporotic fractures. Denosumab is effective in reducing vertebral, nonvertebral, and hip/femoral fractures with an advantage of being administered subcutaneously every six months. The budget impact analysis estimated a reduction in costs for the National Health Service with the introduction of denosumab. Furthermore, the economic analysis demonstrated that denosumab is cost-effective in comparison to oral bisphosphonates and strontium ranelate. Denosumab can be administered in outpatients by involving General Practitioners in the management. Ethical evaluation is positive because of its efficacy and compliance. *Conclusion*. Denosumab could add value in the prevention of osteoporotic fractures.

## 1. Introduction

Osteoporosis, a systemic skeletal disease characterized by low Bone Mass Density (BMD) and microarchitectural deterioration of bone tissue, is a global public health problem currently affecting more than 75 million people worldwide [1]. Eighty percent of people who suffer osteoporosis are women [2]. Osteoporosis causes more than two million fractures annually [3]: the most common are hip/femur and vertebral fractures. Osteoporotic fractures are associated with significant morbidity and mortality [4]. The acute and long-term medical care expenditure associated with osteoporotic fractures was estimated to be $17 billion in 2005 in the United States [5]. In addition to direct medical costs, osteoporosis also results in indirect costs, primarily related to reduced productivity due to disability and premature death [6].

The diagnosis of osteoporosis relies on BMD measurement by means of *T*-score, defined as the number of standard deviations (SDs) from the average BMD of healthy young people. *T*-score measures BMD using central (hip and/or spine) double-energy X-ray scanning [7, 8]. According to World Health Organization (WHO) osteoporosis is diagnosed if *T*-score is ≤−2.5 SD [9].

Due to the reduced BMD, the aim of medical management is to reduce the risk for fracture [10]. Several therapeutic options are available. Among these, a new drug is denosumab, a monoclonal antibody whose administration is characterized by a longer time interval than other drugs [11]. Denosumab is administered through a single subcutaneous injection into the thigh, abdomen, or back of the arm. The recommended dosage is 60 mg once every six months [12].

This study summarizes the results of a Health Technology Assessment (HTA) report on denosumab carried out in 2010 before the drug market access. HTA plays an essential role in modern health care systems by supporting evidence based decision making in health care policy and practice. HTA is concerned with the medical, organizational, economic, and societal consequences of using health technologies within the health system [13] and focuses on the whole value of the technology in the current clinical practice [14].

## 2. Methods

The HTA report about denosumab was carried out by the research group of the Università Cattolica del Sacro Cuore. A multidisciplinary analysis was realized in order to understand the potential of denosumab in the Italian context.

### 2.1. Epidemiological Aspects

PubMed and Ovid SP databases were searched in order to acquire information about osteoporosis pathophysiology, diagnosis, risk factors, epidemiology, and burden of disease. The following keywords were used for the search: “Osteoporosis, Postmenopausal”[Mesh], “Epidemiology/classification”[Mesh], “Epidemiology/diagnosis”[Mesh], “Epidemiology/prevention and control”[Mesh], “Epidemiology/statistics and numerical data”[Mesh], and “Epidemiology/trends”[Mesh]. The Health Search database (i.e., the Italian General Practitioners (GPs) registry) was also looked at in order to draw information about the Italian epidemiological scenario.

### 2.2. Therapeutic Alternatives

Data about efficacy of available antiosteoporotic treatments were retrieved using PubMed, the Cochrane Library, and Ovid SP databases through the keywords “Osteoporosis, Postmenopausal”[Mesh], and “Therapeutics”[Mesh]. According to the hierarchy of evidence, meta-analysis, systematic reviews, and randomized clinical trials (RCTs) were considered; indeed the search was limited to clinical trial and meta-analysis. With respect to RCTs, data from initial pivotal trials were discussed. The drug classes which were taken into consideration were identified according to international and national guidelines for the management of the disease. Ad hoc research strategies were developed for each of the drugs. Literature review was updated to 2010 and Relative Risks (RRs) with 95% confidence intervals (CIs) of hip/femoral, vertebral, and nonvertebral fractures were extracted from the most updated evidence.

### 2.3. Costs of Osteoporosis in Italy

Costs of the disease were estimated with respect to hospitalizations for osteoporotic fractures as the main cost driver [6].

In order to estimate the prevalence of osteoporosis in hospitalized population, discharge rates for osteoporotic fractures were calculated. Population data were obtained from the National Institute for Statistics [15]. Medical records of women aged 45 years or older, discharged from hospital in 2009 for a fracture, and having osteoporosis (ICD-9-CM: 733.0) as principal or secondary diagnosis were taken from National Hospitalizations Database.

Data were stratified by age (45–64, 65–74 and ≥75 years) and by fractures type.

The Diagnosis Related Groups (DRGs) conventional unique tariffs [16] were used to quantify the remuneration as a proxy of the real costs (Euros, 2009). The analysis was conducted from the Italian National Health Service (NHS) perspective.

### 2.4. Budget Impact and Cost-Effectiveness Analysis

A Budget Impact Model (BIM) was used in order to estimate the future impact of the introduction of denosumab on the health care national expenditure. The BIM was made up of three components:

- the demographic model, where the demographic evolution of Italian population was simulated;
- the epidemiological or disease model, where the population of interest (i.e., patients affected by osteoporosis and potentially treated) was reproduced in its evolution in time;
- the technology prediction model, where an estimate of the number of patients administered denosumab and the other available drugs was modelled.

For every year of the time period considered (2010-2013), the BIM took into account the following (the appendix):

- the number of patients treated with each one of the antiosteoporotic available drugs (each with the respective market share),
- the efficacy and complicance to therapy,
- the direct health care costs.

**Figure 1:**
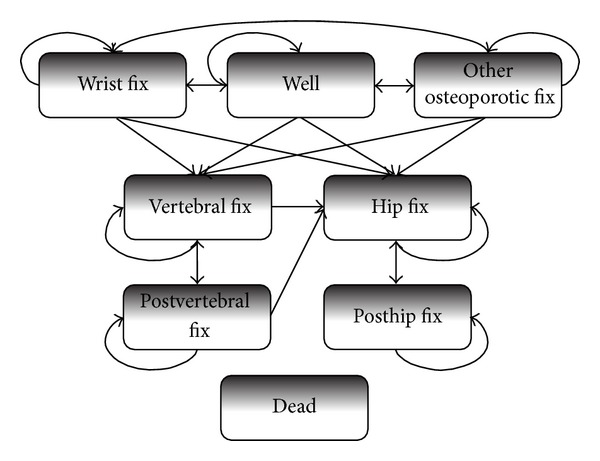
Markov model.

The BIM allowed to estimate costs for Italian NHS with the gradual market access of denosumab. Moreover, a cost-effectiveness analysis was performed from the Italian NHS perspective by using a Markov model which simulated the transition of patients across different health states every six months (Figure 1) [17]. As the economic evaluation was performed from the NHS perspective, only direct medical costs have been included (medications and inpatient, outpatient, and community care). The Markov model was developed in order to assess the cost-effectiveness of denosumab compared with the other alternative treatments. Each cycle was composed of six months. Seven health states have been considered in the analysis: healthy (no fractures), hip/femoral fracture, six months after hip/femoral fracture, vertebral fracture, period following the vertebral wrist fracture, other fractures, and death. According to the transition probabilities from one state to another, the subjects passed from one status to another.

The model was developed by I3 Innovus on Excel software.

Data about costs and efficacy of treatments were the same of the BIM model, while utility data were retrieved from the international literature (the appendix). With respect to efficacy, data were extracted from a document prepared by the National Institute for Clinical Excellence [18] because it was inclusive of data from pivotal trials as well as from their extensions. Efficacy data on the risk of different types of fracture of denosumab relative to placebo were obtained from a phase III randomized placebo-controlled clinical trial (FREEDOM).

With reference to the RR, the details are provided in the appendix. The horizon of the analysis was lifetime and costs and benefits were discounted at 3% per year. Results were reported as incremental cost-effectiveness ratio (ICER), in terms of incremental costs per Quality Adjusted Life Year (QALY) gained.

A probabilistic sensitivity analysis was carried out letting the following variables change simultaneously: unitary costs, fracture risk, compliance, offset time (i.e., the duration of the efficacy after the discontinuation of treatment), mortality rate due to fracture, treatment duration, and utility values.

### 2.5. Organizational Aspects Related to the Use of Denosumab in the Italian Healthcare Context

A literature review was performed in order to analyze organizational impact. In particular, aspects concerning drugs refund, access to treatments and equity, pharmacovigilance, compliance, involvement of GPs, and monitoring of the appropriateness of prescriptions were examined. Data were retrieved using PubMed database and Google search through the keywords “Osteoporosis, Postmenopausal”[Mesh], “Drugs Reimbursement Policies,” “Access to Treatments,” “Equity,” “Pharmacovigilance,” “Compliance,” “General Practitioners,” and “Appropriateness of Prescriptions.”

### 2.6. Ethical Aspects

The ethical issues linked to the utilization of the product were taken into account through a framework including epistemological data, anthropologic reference, and ethical evaluation.

## 3. Results

### 3.1. Epidemiological Aspects

Osteoporosis is a leading cause of morbidity and mortality in elderly people, especially women, worldwide. According to the WHO, osteoporosis affects more than 75 million people in the United States, Europe, and Japan [19]. In the United States and the European Union about 30% of postmenopausal women are affected, and it is estimated that more than 40% have an osteoporotic fracture during their lives [20].

It has been estimated that the worldwide incidence of hip/femoral fractures will rise both in men and women [21]. Furthermore, even though age-adjusted incidence rates remained stable, the absolute number of hip/femoral fractures worldwide would increase reaching 6.26 million in 2050 [22].

In Italy the frequency of the disease has been studied through the Epidemiological Study On The Prevalence of Osteoporosis (ESOPO) [23], conducted in 2000. The study showed a prevalence of osteoporosis of 22.8% among women aged between 40 and 79 years and almost 50% considering only women over the age of 70 years. Nowadays, it is estimated that osteoporotic women in Italy are about four million and by 2025 they will be around five million [23, 24]. The prevalence of osteoporotic fractures is growing. Indeed, there are about 70,000 women hospitalized for hip/femoral fracture yearly in Italy and the incidence of hip/femoral fractures increased by 28% in women over 74 years between 2000 and 2005 [25, 26]. The query of Health Search database for the period 2006–2008 released a steady incidence of osteoporotic fractures. Furthermore it demonstrated an increased annual incidence in elderly people (0.74–0.85%, 1.47–1.51%, and 1.87–2.20% in women aged 65–74, 75–84, and over 84 years, resp.).

In conclusion, osteoporosis represents an emerging condition in our country and around the world, mainly due to worldwide ageing of population.

### 3.2. Therapeutic Alternatives

According to the Italian guidelines [10] the following drugs are used in the medical management of osteoporosis:

- bisphosphonates avoid bone resorption and include etidronate, alendronate, and risedronate which are given orally, ibandronate which is administered both orally and intravenously, zoledronate, given intravenously, and clodronate for intramuscular or oral administration;
- strontium ranelate which works both stimulating osteogenesis and avoiding osteoresorption;
- analogues of parathormone (PTH 1–34—teriparatide—and PTH 1–84);
- selective estrogens receptor modulators (SERM; i.e., raloxifene);
- hormone replacement therapy (HRT);
- denosumab.

Therefore, drugs used in osteoporosis may be classified in antiresorptive and osteogenic: in the first group bisphosphonates, SERM, and HRT may be listed, while in the second group parathormone analogues are found. Strontium ranelate works through both mechanisms.

All drug classes have been demonstrated to be effective in reducing vertebral fractures when given together to vitamin D and calcium and are recommended for the treatment of postmenopausal osteoporosis.

The review of the literature yielded several meta-analyses and pivotal RCTs which have proved the efficacy of the different drugs. According to the evidence reported in Table 1, parathormone analogues as well as several bisphosphonates may maximize the reduction in vertebral and nonvertebral fractures till 70%. Prevention of hip/femoral fractures would be maximized with bisphosphonates. Anyhow, because of high costs of parathormone analogues, they are not recommended as first line treatment [10].

Denosumab is a fully human monoclonal antibody of IgG2 subtype produced in Chinese hamster ovary cells, inhibiting RANK ligand (RANKL). Inhibition of RANKL is one of the possible interventions able to interfere with conditions with increased bone resorption. RANKL production is increased when estrogen is decreased, as after menopause and in conditions of hormone ablation, leading to an increased bone resorption. This suggests a change in the ratio of RANKL and counterbalancing decoy receptor osteoprotegerin that promotes bone resorption [27].

Denosumab, at a dose of 60 mg subcutaneous injection every six months, seems to be an effective treatment for postmenopausal osteoporosis as it is able to reduce the incidence of vertebral, nonvertebral, and hip/femoral fractures. In particular, the FREEDOM study is an international, multicenter, randomized, double-blind, placebo-controlled trial conducted to evaluate the efficacy and safety of denosumab in reducing the incidence of new vertebral (primary endpoint) and spine and hip/femoral (secondary endpoints) fractures in women with postmenopausal osteoporosis [28]. A total number of 7,868 women aged 60 to 90 years having a *T*-score <−2.5 and ≥−4.0 were enrolled in the study. They were randomly assigned to receive either 60 mg of denosumab or placebo for 36 months. As compared with placebo, denosumab reduced the risk of new radiographic vertebral fractures, with a cumulative incidence of 2.3% in the denosumab group versus 7.2% in the placebo group (RR 0.32; 95% CI 0.26–0.41). Denosumab reduced the risk of hip/femoral fracture, with a cumulative incidence of 0.7% versus 1.2% in the placebo group (hazard ratio (HR) 0.60; 95% CI 0.37–0.97). Denosumab also reduced the risk for nonvertebral fractures, with a cumulative incidence of 6.5% versus 8.0% in the placebo group (HR 0.80; 95% CI 0.67–0.95). There was no increase in the risk of cancer, infection, cardiovascular disease, delayed fracture healing, or hypocalcemia, and there were no cases of osteonecrosis of the jaw and no adverse reactions to the injection. The majority of adverse events were mild or moderate in severity, having transient duration, and not considered related to administration of denosumab [28].

The efficacy and safety of denosumab compared to alendronate have been also evaluated in two phase III noninferiority clinical trials for a period of one year (DECIDED and STAND study) [29, 30].

In the first study, 1,189 postmenopausal women with a *T*-score ≤−2.0 were randomized to receive subcutaneous denosumab injections plus oral placebo weekly (*n* = 594) or oral alendronate weekly (70 mg) plus subcutaneous placebo injections (*n* = 595). The authors evidenced that denosumab treatment led to significantly larger gains in BMD and a greater reduction of bone turnover markers compared with alendronate. The overall safety profile was similar for both treatments [29].

In the second one, 504 postmenopausal women, after a 1-month run-in period during which all of them received open-label, branded alendronate 70 mg once weekly, were randomly assigned to receive subcutaneous denosumab injections 60 mg once every six months or to continue receiving branded alendronate 70 mg once weekly. Transition to denosumab produced greater increases in BMD at all measured skeletal sites and a greater reduction in bone turnover. Adverse events and serious adverse events were balanced between groups. No clinical hypocalcaemia was reported [30].

### 3.3. Costs of Osteoporosis in Italy

In Italy, the discharge rate for osteoporotic fractures was 35.60 per 100,000 in women aged 45 years or older; this value increased with age for all types of fractures and reached the highest value for hip/femoral fractures (17.11 per 100,000 women aged 45 years or over).

Table 2 shows the annual direct costs for hospitalization among women in Italy. The annual mean cost for hospitalization amounted to €2,241.96. The main cost driver was represented by hip/femoral fracture (around €14 million).

### 3.4. Budget Impact and Cost-Effectiveness Analysis

According to the BIM and the market and demographic forecasting, the number of patients given denosumab will increase during the three-year horizon from 60,000 patients managed with the drug in the first year to 150,000 in the third year. Taking into consideration data about efficacy and compliance, an absolute reduction of 93 cases of hip/femoral fractures is expected in the first year and of 275 in the third year. Similarly, the number of vertebral fractures would decrease of 135 in the first year and 372 in the third year. Savings are shown in Table 3 together with absolute number of fractures. In particular, in Table 3, for each of the considered years (from 2010 to 2013) the following are reported:

- costs related to the treatment of hip/femoral and vertebral fractures in the group treated with denosumab;
- number of hip/femoral and vertebral fractures avoided by treating the subjects with denosumab;
- related savings.

As far as the nonvertebral fractures are concerned, there would be an absolute reduction of 54 and 138, respectively, in the first and third years for a saving of €231,000 and €593,000. The majority of costs avoided are due to hospitalizations accounting for 62.3–82.4% of total costs.

With respect to drugs expenditure, the model yielded a future increase in costs independently by the introduction of denosumab mainly due to the demographic evolution. However, because of denosumab higher adherence and efficacy, there would be a whole saving for the Italian NHS. The saving would be mainly driven by the replacement of high costs drugs with denosumab, as reported in Table 4. In particular, costs and outcomes in Table 4 are intended per year. For each of the considered alternatives, the yearly related costs are reported across the four years considered in the analysis in both scenarios: with denosumab versus without denosumab.

Table 5 shows the results of the budget impact analysis for each of the following costs voices considered:

- medications;
- inpatient care;
- outpatient care;
- community care.

Furthermore, the total results (expressed in thousands €) are reported. In particular, in the BIM, two scenarios are considered:

- scenario with denosumab;
- scenario without denosumab.

By comparing these two scenarios, it is possible to state that the introduction of denosumab within the Italian health care setting would lead to significant savings from 2011 to 2013 (from €5,190,000 to €14,904,000).

The cost-saving profile is preserved also if an increase of costs is considered as well as a decrease in efficacy and compliance.

With respect to cost-effectiveness analysis, considering a study population 65 years old with a *T*-score <−4 SD, denosumab was shown to be cost-effective in comparison to risedronate, generic and branded alendronate, ibandronate, and strontium ranelate (Table 6). In Table 6, total costs, QALYs, and ICERs are reported for each of the alternatives considered. QALYs measures have been calculated by multiplying each year gained by the related utility. The utility values have been collected from the literature (the appendix, Table 8). ICERs have been calculated by using the following formula:

(1) $$\begin{array}{c} \text{ICER}=\frac{\Delta \text{Costs}}{\Delta \text{QALYs}}. \end{array}$$

Taking into consideration a threshold of €30,000, denosumab was demonstrated to be cost-effective with a probability of 85% versus risedronate and 95% versus ibandronate and strontium ranelate. In comparison to alendronate denosumab was shown to be cost-effective with a probability of around 65%.

### 3.5. Organizational Aspects Related to the Use of Denosumab in the Italian Healthcare Context

Osteoporosis represents a social as well as an economic priority, due to the progressive ageing of the population [3, 31]. Therefore, given the suboptimal adherence to the existing treatments (≥80% just in 51.2% of cases) [32], it seems appropriate to introduce innovative therapies, such as denosumab, allowing a more effective management. It is also necessary to consider the strong inequalities in the distribution and access to treatments due to the heterogeneous distribution of innovative drugs in Italy [33]. Concerning osteoporosis, according to the *Nota AIFA n. 79* (Nota AIFA is a mandatory guideline provided by the Italian Drug Agency (whose acronym is AIFA) indicating the criteria for the reimbursement of drugs costs by the Italian NHS. It is also a tool aimed at monitoring and controlling appropriateness of prescriptions and pharmaceutical expenditure.) [34], drugs costs are refunded by the Italian NHS when some specific criteria related to the presence in the patient of some risk conditions are satisfied. The appropriateness of prescription of denosumab, as a new drug available for treating osteoporosis, could be indeed regulated by the same *Nota AIFA n. 79*. This drug, considered its characteristics in terms of both way of administering and favourable safety profile, lends itself to be used in outpatient care [30, 35]. Therefore GPs should be involved in the management of osteoporosis therapy as well as in prevention. GPs in fact, unlike specialists, provide care for a considerable number of patients (different by gender, age, ethnicity, occupation, and lifestyle) and have a comprehensive vision of the assisted people, taking into account their general clinical condition, family history, psychological and physical characteristics, and compliance to therapy [35].

### 3.6. Ethical Aspects

The efficacy of denosumab in terms of increased BMD and reduced incidence of osteoporotic fractures [28–30, 36] lies in the distribution and use of this drug. However, on the other hand, caution is required in controlling adverse effects [28, 37–41] and promoting patient's quality of life. A proper use of denosumab requires decision makers to provide each patient personalized interviews in order to take into account each specific situation, check the possibility and feasibility of an equal access to the drug by all patients with osteoporosis, ask GPs to pay a particular attention in monitoring the effectiveness of the drug and in reporting adverse events [35]. The scientific community has to be engaged in the organization of further RCTs in order to gather further evidence on denosumab with reference to efficacy, safety, and compliance [42]. In particular, studies investigating long-term effects of denosumab should be encouraged in order to better assess its safety. Furthermore, head-to-head investigation should be promoted instead of studies controlled with placebo, and hard endpoints, instead of BMD, should be considered for testing the efficacy of the drug.

## 4. Discussion

This HTA report supported the value of denosumab by demonstrating its efficacy and cost-effectiveness in comparison to the available alternatives and the feasibility of medical management because of the therapy schedule. Other agencies such as the National Institute of Health and Clinical Excellence (NICE) and the Canadian Agency for Drugs and Technology in Health (CADTH) addressed the use of denosumab in preventing osteoporotic fractures with respect to clinical effectiveness, safety, and cost-effectiveness. Both of them assessed that denosumab may be used in postmenopausal women with osteoporosis who have switched from oral bisphosphonates (alendronate, risedronate, or etidronate) because of its safety and effectiveness [43, 44]. Unlike these reports, our analysis took into account the comparison to oral bisphosphonates because at the time of the assessment denosumab had not accessed the market yet. Therefore our goal was to define also the potential value of denosumab in comparison to drugs which are recommended as first line treatment [10]. Moreover, our study compared denosumab to strontium ranelate which was suggested by NICE as a drug to be used in primary and secondary prevention of osteoporosis fragility fractures in postmenopausal women who are unable to comply with oral bisphosphonates [45, 46]. According to the NICE evaluation, denosumab dominated strontium ranelate in that it was more effective and less costly. Our results showed that denosumab was highly cost-effective with an ICER of €69/QALY and had a 95% probability of being considered cost-effective, by considering a willingness to pay threshold/QALY of €30,000.

According to these results, denosumab may be considered a good therapeutic alternative to strontium ranelate in preventing osteoporotic fragility fractures. With respect to oral bisphosphonates, our analysis yielded that denosumab is cost-effective too: this result may be due to the higher compliance level which was hypothesized for denosumab and is also supported by other pieces of evidence [44]. Compliance is a relevant element in determining efficacy and effectiveness of treatments and could be the leading driver of osteoporotic fractures decrease. Furthermore, from a public health perspective a higher compliance could make easier the management of patients and reduce its costs.

The present study has some limitations concerning the data input of the clinical and economic forecasting and the estimates of costs related to osteoporotic fractures. In fact, data about efficacy were mainly drawn from meta-analyses and RCTs which encompassed a placebo group and were not head-to-head studies. As far as utilities are concerned, data were extracted from the international literature because of the lack of national evidence. With respect to BIM model and cost-effectiveness analysis, the market shares were just hypothesized and indirect costs (mostly related to caregiving) were not considered. Finally a possible underestimation of direct costs of osteoporotic fractures should be considered. In fact, we only took into account costs of hospitalization, as Italian outpatient activity data are not available. Notwithstanding these concerns, it should be stated that the HTA is a comprehensive approach in the evaluation of health technologies. Therefore, the value of denosumab in the prevention of osteoporotic fracture has been highlighted in depth.

## 5. Conclusion

The population ageing will lead to an increase in prevalence of osteoporotic women at risk for fragility fractures. In this light, prevention as well as therapeutical interventions will play an important role and will represent a public health priority. Notwithstanding, decision making process should be supported by evidence. The HTA, thanks to its thorough approach to the evaluation of health technologies, may be quite important to support decision makers with respect to issues related to the introduction, recommendation, use, and reimbursement of drugs used to prevent fragility fractures. In particular, with respect to denosumab, the results of the HTA showed that it is effective, cost-effective, and easy to be managed. Therefore, its use should be recommended in the Italian context.

## Highlights

- Osteoporosis represents a public health issue mainly affecting postmenopausal women.
- Denosumab is a drug able to reduce the risk of osteoporotic fractures.
- Denosumab is cost-effective versus alendronate, ibandronate, and strontium ranelate.
- This drug may be administered in outpatient care given its characteristics.
- The Health Technology Assessment report supports the use of the drug.

## Figures and Tables

**Table 1:** Results of literature review about efficacy of antiosteoporotic drugs.

	First author and year	Study design	Study population	Outcome	*N*	Results		
						Vertebral fractures	Nonvertebral fractures	Hip/femoral fractures
Alendronate	Wells et al. 2008 [49]	Cochrane review	Postmenopausal women	Osteoporotic fractures after ≥1 year of follow-up	12,068 (4,576 in primary prevention)	Reduction of 45% (33–55%) (primary prevention)	Reduction of 16% (6–26%)	Reduction of 40% (7–60%) of femoral fractures and of 21% (n.s.) of hip fractures
	Black et al. 2000 (FIT) [50]	RCT	Women in menopause since at least 2 years	Fractures after 3/4 years of treatment	6,459	Single vertebral fractures: reduction of 47% (32–59%). Multiple vertebral fractures: reduction of 90% (78–95%)	Reduction of 19% (n.s.)	Reduction of 51% (1–77%)
	Cummings et al. 1998 (FIT) [51]	RCT	Women in menopause since at least 2 years	Fractures after 4 years of treatment	4,432	One or more vertebral fractures: reduction of 44% (39–80%) Two or more vertebral fractures: reduction of 60% (n.s.)	Reduction of 12% (n.s.)	Reduction of 21% (n.s.)
	Black et al. 1996 (FIT) [52]	RCT	Women in menopause since at least 2 years	Fractures after 24 and 36 months of treatment	2,027	One or more morphometric vertebral fractures: reduction of 47% (32–59%). Two or more morphometric vertebral fractures: reduction of 90% (78–95%)	Reduction of 20% (n.s.)	Reduction of 48% of hip fractures (13–69%)
	Liberman et al. 1995 [53]	RCT	Women in menopause since at least 5 years	Osteoporotic fractures after 3 years of treatment	881	Reduction of 48% (5–72%)	—	—

Risedronate	Wells et al. 2008 [54]	Cochrane review	Postmenopausal women	Osteoporotic fractures	14,049	Reduction of 27% (23–49%) (primary and secondary prevention)	Reduction of 20% (10–28%) (primary and secondary prevention)	—
	Harris et al. 1999 (VERT) [55]	RCT	Women in menopause since at least 5 years, aged <85 years and with ≥2 vertebral fractures	Osteoporotic fractures after 1 and 3 years of treatment	2,458	First year of treatment: reduction of 46% (9–68%) with risedronate 2,5 mg and of 65% (38–81%) with risedronate 5 mg. After 3 years of treatment: reduction of 41% (18–57%)	After 3 years of treatment: reduction of 40% (6–61%)	—
	Reginster et al. 2000 (VERT) [56]	RCT	Women in menopause since at least 5 years, aged <85 years and with ≥2 vertebral fractures	Osteoporotic fractures after 3 years of treatment	1,226	Reduction of 49% (27–64%)	Reduction of 33% (n.s.)	
	McClung et al. 2001 (HIP) [57]	RCT	Postmenopausal women aged 70–79 years	Osteoporotic fractures after 3 years of treatment	5,445	—	—	Reduction of 40% (10–60%)

Ibandronate	Harris et al. 2008 [58]	Meta-analysis	BONE, IV Fracture Prevention, MOBILE, and DIVA studies	Nonvertebral and clinical fractures	8,710	—	Reduction of 29.9%	—
	Chesnut III et al. 2004 (BONE) [59]	RCT	Women in menopause since at least 5 years, aged 55–80 years, with 1–4 previous vertebral fractures and a *T*-score of −2.0 to −5.0 in at least one vertebral site	New morphometric vertebral fractures at 3 years of treatment	2,946	Reduction of 62% (41–75%) for daily dose and of 50% (26–66%) for intermittent dose	Nonsignificant results	—

Zolendronate	Black et al. 2007 (HORIZON) [60]	RCT	Women aged 65–89 years with a *T*-score ≤ 2,5 (with or without vertebral fractures) or *T*-score ≤ 1,5 and at least 2 vertebral fractures	Osteoporotic fractures after 3 years of treatment	7,765	Reduction of 70% (62–76%)	Reduction of 25% (13–36%)	Reduction of 41% (17–58%)

Strontium ranelate	Meunier et al. 2004 (SOTI) [61]	RCT	Women ≥ 50 years with ≥1 osteoporotic vertebral fractures	Vertebral fractures after 3 years of treatment	1,649	Reduction of 49% (26–64%) after 1 year and of 41% (27–52%) after 3 years	—	—
	Reginster et al. 2005 (TROPOS) [62]	RCT	Women ≥ 74 years (or aged 70–74 with at least another risk factor for fracture)	Vertebral and nonvertebral fractures	5,091	Reduction of 45% (23–61%) after 1 year and of 39% (27–49%) after 3 years	Reduction of 16% (0.5–29.8%). Reduction of 19% of major fractures (2–34%)	

PTH 1-34 and 1-84	Vestergaard et al. 2007 [63]	Meta-analysis	Postmenopausal women	Osteoporotic fractures	4,155	Reduction of 63% (52–72%)	Reduction of 38% (18–54%)	—

PTH 1-34	Neer et al. 2001 (FPT) [64]	RCT	Women in menopause since at least 5 years with ≥2 mild vertebral fractures or 1 moderate vertebral fracture	Osteoporotic fractures after 2 years of treatment	1,637	PTH 1-34, 20 *µ*g/die: reduction of 65% (45–78%). PTH 1-34, 40 *µ*g/die: reduction of 69% (50–81%)	PTH 1-34, 20 µg/die: reduction of 53% (12–75%). PTH 1-34, 40 µg/die: reduction of 54% (14–75%)	—

PTH 1-84	Greenspan et al. 2007 (TOP) [65]	RCT	Women aged 45–54 years with *T*-score ≤ −3 and no previous fractures or *T*-score ≤ −2,5 and 1–4 previous fractures, and women ≥ 55 years with *T*-score ≤−2.5 and no previous fractures or *T*-score ≤ −2 and 1–4 previous fractures	New osteoporotic fractures or worsening of previous osteoporotic fractures after 18 months of treatment	2,532	Reduction of 58% (28–76%)	Nonsignificant results	—

SERM	Cranney et al. 2002 [66]	Meta-analysis	Postmenopausal women	Vertebral and non-vertebral fracture	7,848	Reduction of 40% (30–50%) with a dosage ≥60 mg/die	Nonsignificant results	—
	Ettinger et al. 1999 (MORE) [67]	RCT	Women in menopause since at least 2 years, aged < 80 years with a *T*-score ≤ −2,5 or previous vertebral fractures	Vertebral and non-vertebral fractures	7,705	Reduction of 30% (20–50%) with 60 mg/die and 50% (30–60%) with 120 mg/die	Non significant results	—

TOS	Torgerson and Bell-Syer 2001 [68]	Meta-analysis	Postmenopausal women	Vertebral fractures	6,723	Reduction of 33% (2–55%). Reduction of 53% (11–75%) in women with previous osteoporosis and of 37% (4–59%) in women > 60 years	—	—
	Cauley et al. 2003 (WHI) [69]	RCT	Women aged 50–79 years with a BMD ≥ 3 than age-specific mean	Vertebral, hip, and other osteoporotic fractures	16,608	—	Reduction of 25% (17–32%)	Reduction of 33% (4–53%)

n.s.: not significant; BONE: oral iBandronate Osteoporosis vertebral fracture trial in North America and Europe; FIT: Fracture Intervention Trial; FPT: Fracture Prevention Trial; HIP: Hip Intervention Program; HORIZON: Health Outcomes and Reduced Incidence with Zoledronic Acid ONce yearly; MORE: Multiple Outcomes of Raloxifene Evaluation; SOTI: Spinal Osteoporosis Therapeutic Intervention; TROPOS: TReatment Of Peripheral OSteoporosis; TOP: Treatment of Osteoporosis with Parathyroid hormone; VERT: Vertebral Efficacy with Risedronate Therapy; WHI: Women's Health Initiative.

**Table 2:** Annual direct costs for hospitalization in Italy (*€* 2009).

Fractures type	Number of admissions	Total mean direct costs (*€*)
Hip and femur	4,653	14,077,146.05
Vertebral	2,318	2,549,459.76
Other	3,254	6,297,477.79

Total	10,225	22,924,083.60

**Table 3:** Number of fractures occurring in patients given different treatments and pertaining costs.

	2010	2011	2012	2013
Hip/femoral fractures (alternatives)	36,343	37,142	37,971	38,657
Hip/femoral fractures (denosumab)	36,343	37,049	37,808	38,382
Hip/femoral fractures avoided with denosumab	—	93	163	275
Saving (€)	—	860,000	2,042,000	3,788,000
Vertebral fractures (alternatives)	21,487	21,506	21,612	21,640
Vertebral fractures (denosumab)	21,487	21,370	21,387	21,268
Vertebral fractures avoided with denosumab	—	136	225	372
Saving (€)	—	333,000	653,000	1,139,000

**Table 4:** Costs of medications in thousands € stratified by year and by scenario (with or without denosumab).

	2010		2011		2012		2013	
	Without denosumab	With denosumab	Without denosumab	With denosumab	Without denosumab	With denosumab	Without denosumab	With denosumab
Alendronate (Fosamax)	27,859	27,859	26,359	26,359	26,114	26,114	25,959	25,959
Alendronate plus cholecalciferol (Fosavance)	48,040	48,040	49,413	49,092	48,432	47,933	49,513	48,713
Generic alendronate	72,561	72,561	76,132	76,132	80,274	80,274	83,254	83,254
Risedronate (Actonel)	90,527	90,527	57,872	57,872	60,843	60,843	63,888	63,888
Zoledronate (Actonel)	2,940	2,940	4,962	4,962	5,263	5,263	5,421	5,421
Ibandronate oral (Bonviva)	43,313	43,313	51,187	44,209	54,375	43,527	61,116	43,725
Raloxifene (Evista)	6,847	6,847	7,457	7,457	7,688	7,688	7,919	7,919
Strontium ranelate (Protelos)	58,074	58,074	78,835	56,736	93,214	58,858	111,307	56,232
Teriparatide	50,701	50,701	54,415	54,415	57,001	57,001	60,584	60,584
PTH	10,244	10,244	11,070	11,070	11,243	11,243	11,581	11,581

**Table 5:** Budget impact in thousands € with denosumab.

	2010	2011	2012	2013
Medications	—	−3,766	−5,854	−9,385
Inpatient care	—	−1,174	−2,025	−3,383
Outpatient care	—	−250	−420	−689
Community care	—	—	−624	−1,447

Total	—	−5,190	−8,923	−14,904

**Table 6:** Results of cost-effectiveness analysis.

		Denosumab	Alternative	Difference	ICER (€/QALY)
Risedronate	Total costs	22,399	21,819	579	10,302
	QALYs	10.46	10.40	0.06	

Generic alendronate	Total costs	22,399	21,621	778	18,047
	QALYs	10.46	10.41	0.04	

Branded alendronate	Total costs	22,399	21,661	738	17,133
	QALYs	10.46	10.41	0.04	

Ibandronate	Total costs	22,399	22,238	161	2,158
	QALYs	10.46	10.38	0.07	

Strontium ranelate	Total costs	22,399	22,394	5	69
	QALYs	10.46	10.39	0.07	

**Table 7:** Data input for BIM and cost-effectiveness analysis: efficacy in terms of prevention of different osteoporotic fractures and compliance to the antiosteoporotic treatments available.

	Efficacy [18, 28] (%)			Compliance^1^ [17] (%)
	Hip/femoral	Vertebral	Nonhip nonvertebral	
Denosumab	40	68	20	85
Alendronate	38	44	17	60
Risedronate	26	36	25	60
Zoledronate	41	70	24	60 (generic), 100 (Aclasta)
Ibandronate	0	49	0	60
Raloxifene	0	36	10	60
Strontium ranelate	15	38	9	60
Teriparatide	75	65	60	60
PTH	35	61	3	60

^1^: As there is no data available for Italy, data reported in the table are based only on assumptions.

**Table 8:** Unitary costs and utility values used in the economic model.

	Unitary costs (mean direct medical costs, €) [48, 70–72]	Utilities (first year after fracture; second and following years after fracture)
Hip fracture	8.206	0.700 [73]; 0.800 [73]
Vertebral fracture	2.476	0.590 [73]; 0.929 [74]
Other fractures		0.956 [73] wrist, 0.902 [70] other fractures
(i) Pelvis and other femoral fractures	4.575	
(ii) Forearm	2.831	
(iii) Ribs and sternum	1.022	
(iv) Scapula and clavicle	2.962	
(v) Proximal humerus and humeral shaft	4.575	
(vi) Tibia and fibula	4.929	

**Table 9:** Market shares for each therapeutic alternative.

Product	2010	2011	2012	2013
Denosumab	0.0%	2.3%	5.2%	8.0%
Fosamax	9.7%	8.6%	8.2%	7.7%
Fosavance	15.9%	15.4%	14.2%	13.7%
Generic alendronate	27.7%	27.3%	27.4%	27.0%
Actonel	24.3%	24.6%	24.4%	24.3%
Aclasta	0.3%	0.4%	0.4%	0.4%
Bonviva oral	9.3%	9.0%	8.3%	7.9%
Evista	1.7%	1.7%	1.7%	1.7%
Protelos	9.8%	9.3%	8.9%	8.1%
Miacalcic	0.2%	0.2%	0.1%	0.1%
Teriparatide	0.9%	0.9%	0.9%	0.9%
PTH	0.2%	0.2%	0.2%	0.2%

Total	100%	100%	100%	100%

## Appendix

In this section input data for the BIM and cost-effectiveness analysis and the formula used to calculate the reduction risk of fracture are reported.

With reference to the baseline risk, the incidence of hip/femoral fractures was obtained from the publication by Piscitelli et al. [26].

As no reliable data are available for the incidence of clinically diagnosed vertebral fractures in Italy, the approach taken by Stevenson et al. for the NICE Health Technology Assessment in the UK [47] was used: the incidence of vertebral fractures was estimated by applying the ratios of vertebral to hip fractures observed in Sweden [48] to the incidence of fracture observed in Italy. The same approach was used for nonhip nonvertebral fractures. The distribution by site can be assumed to be constant across European countries, as confirmed by experts.

In Table 7, data about efficacy of antiosteoporotic drugs, taken by the NICE meta-analysis, (NICE. Systematic reviews of clinical effectiveness prepared for the guideline osteoporosis: assessment of fracture risk and the prevention of osteoporotic fractures in individuals at high risk 2008. http://www.nice.org.uk/nicemedia/pdf/OsteoporosisEvidenceReviews190908.pdf, accessed on December 16, 2009), which were considered in our analysis and compliance to therapy are reported.

In Table 8 data about unitary costs of fractures and utilities are enclosed.

Unitary costs of different treatments were extracted from “AA VV. L'informatore farmaceutico 2010. Elsevier Masson Eds. 2010. pp. 2199 ISBN: 978-88-214-3185-2.” The price for denosumab was only hypothesized, as it was not yet defined by Italian Medicines Agency (AIFA: Agenzia Italiana del Farmaco) when our economic model was carried out and equal to €507 for a year of therapy.

The efficacy was corrected by the assumed compliance for each of the alternatives treatments.

The equation to calculate the relative risk (RR) of fracture given a specific target population can also be written as

(A.1) RRfx,target=RRfx/sd−Zscore=exp⁡[ln⁡(RRfx/sd)∗−Zscore].

The *Z*-score is normally distributed. The exponentiated *Z*-score (exp⁡^(*Z*_score_)^) is exponentially distributed. A statistical correction factor using a truncated normal distribution was introduced in this equation. The reason for this is that when the expectation of *Z*-score is imputed in the exponentiated *Z*-score, the results are unequal to the expectation of the exponentiated *Z*-score. The statistical correction factor was approximated by

(A.2) $$\begin{array}{c} \exp\left[-\frac{{\left(\ln\left(\text{R}{\text{R}}_{fx/sd}\right)\right)}^{2}}{2}\right]. \end{array}$$

In order to calculate the relative risk of fracture for a specific target population, the above equations are multiplied, yielding

(A.3) RRfx,target=exp⁡[[ln⁡(RRfx/sd)∗−Zscore]    −[(ln⁡⁡(RRfx/sd))22]].

With reference to market shares used to develop the BIM analysis, Table 9 shows the percentages for each alternative and year, provided by the manufacturer.
